# A new method for measuring the neutron lifetime using an *in situ* neutron detector

**DOI:** 10.1063/1.4983578

**Published:** 2017-05-30

**Authors:** C. L. Morris, E. R. Adamek, L. J. Broussard, N. B. Callahan, S. M. Clayton, C. Cude-Woods, S. A. Currie, X. Ding, W. Fox, K. P. Hickerson, M. A. Hoffbauer, A. T. Holley, A. Komives, C.-Y. Liu, M. Makela, R. W. Pattie, J. Ramsey, D. J. Salvat, A. Saunders, S. J. Seestrom, E. I. Sharapov, S. K. Sjue, Z. Tang, J. Vanderwerp, B. Vogelaar, P. L. Walstrom, Z. Wang, Wanchun Wei, J. W. Wexler, T. L. Womack, A. R. Young, B. A. Zeck

**Affiliations:** 1 Los Alamos National Laboratory, Los Alamos, New Mexico 87545, USA; 2 Department of Physics, Indiana University, Bloomington, Indiana 47408, USA; 3 Oak Ridge National Laboratory, Oak Ridge, Tennessee 37831, USA; 4 Triangle Universities Nuclear Laboratory, North Carolina State University, Raleigh, North Carolina 27695, USA; 5 Department of Physics, Virginia Polytechnic Institute and State University, Blacksburg, Virginia 24061, USA; 6 California Institute of Technology, Pasadena, California 91125, USA; 7 Department of Physics, Tennessee Tech University, Cookeville, Tennessee 38505, USA; 8 Department of Physics, DePauw University, Greencastle Indiana 46135-0037, USA; 9 Department of Physics, University of Washington, Seattle, Washington 98195-1560, USA; 10 Joint Institute for Nuclear Research, Dubna, Moscow Region 141980, Russia

## Abstract

In this paper, we describe a new method for measuring surviving neutrons in neutron lifetime measurements using bottled ultracold neutrons (UCN), which provides better characterization of systematic uncertainties and enables higher precision than previous measurement techniques. An active detector that can be lowered into the trap has been used to measure the neutron distribution as a function of height and measure the influence of marginally trapped UCN on the neutron lifetime measurement. In addition, measurements have demonstrated phase-space evolution and its effect on the lifetime measurement.

## INTRODUCTION

Two different techniques have been used to measure the neutron lifetime: by measuring the decay rate in a cold neutron beam using a Penning trap to capture and count resultant protons and by measuring the survival of neutrons after storage using trapped ultracold neutrons (UCN).^1^ The most precise measurements from a material bottle (878.5 ± 0.8 s^2^) and cold beam measurement (887.7 ± 2.2 s^3^) disagree by 3.9 standard deviations. The probability of both measurements being consistent with the neutron lifetime is about 1 × 10^−4^.

Because the neutron lifetime controls weak reaction rates for n ↔ p at freeze out in the early universe and therefore directly affects the ^4^He abundance, the uncertainty in big bang nucleosynthesis (BBN) predictions of the ^4^He abundance is dominated by the uncertainty in the neutron lifetime.^4^ Resolving the discrepancy between beam and bottle lifetime results and improving the precision to the sub-one second level is the key to improving BBN predictions of primordial elemental abundances. Comparison of the predicted abundances with astrophysical measurements provides additional tests of SM physics.

The unitarity of the Cabbibo-Kobayashi-Maskawa (CKM) matrix provides a test of the standard model sensitive to a host of new physics beyond the standard model.^5^ The best test comes from the first row of the CKM matrix because of precise measurements of V_ud_ resulting from an analysis of super-allowed nuclear beta decays that dominate the unitarity sum and the uncertainty.^6^ Measurements at the level of a few 10^−4^ of the neutron lifetime, τ_n_, and about 10^−3^ in the neutron β asymmetry,^7–9^ A, can provide a determination of V_ud_ free of the nuclear structure corrections that contribute the precision that can be obtained from super-allowed beta decay and are somewhat controversial.^10^

UCN experiments have traditionally used material bottles for neutron storage. In these experiments, neutrons are loaded into a bottle and the remaining neutrons are unloaded and counted after a variable storage time. The spectral dependence of neutron up-scatter and absorption leads to softening of the neutron energy/velocity spectrum as a function of storage time. Consequently, the detection efficiency and unloading time become storage-time dependent. Uncertainty in the systematic corrections associated with spectral and phase space evolution forms an important contribution to the total lifetime uncertainty in many of the bottle measurements.^2,11–13^ Huffman *et al.* have attempted to reduce these systematic uncertainties by using a magnetic quadrupole trap to eliminate material walls and by measuring neutron decays *in situ* by detecting the electrons from neutron decay in the superfluid helium that was also used to produce the UCN using “super-thermal” production in a cold neutron beam.^14^ Unfortunately, poor signal-to-noise ratio and other systematic uncertainties limited the precision of this measurement to several hundred seconds. Serebrov *et al.*^2^ were able to reduce these effects by using a larger trap to reduce the wall collision rate and lower surface temperature to reduce the loss per wall collision, and have published the smallest uncertainty for the neutron lifetime to date. Still, the largest corrections to the measured lifetime in previous experiments were due to loss on material surfaces. In these experiments, this correction was controlled by changing the surface to volume ratio and extrapolating the loss rate to zero, an extrapolation of >5 s for the experiment^2^ of Serebrov *et al.* and larger for previous experiments.^11,12^

Ezhov *et al.*^15–17^ have demonstrated UCN storage in a 20-pole axisymmetric magnetic bottle made of permanent magnets and have reported a preliminary lifetime, τ_n_ = 878.3 ± 1.9 s, in agreement with the Serebrov measurement. The current experiment (UCNτ) aims to reduce systematic uncertainties encountered in these experiments by storing the neutrons in an asymmetric magneto-gravitational trap^18,19^ that eliminates wall losses, limits the population of long-lived quasi-bound UCN, and detects the neutrons *in situ* at the end of the storage time. In this paper we describe the *in situ* detector and demonstrate that shorter counting times can be achieved with this method when compared to previous bottle measurements (viz., the time it takes to “empty” the trap). Further, we investigate the presence of long lived phase space evolution in our trap, a potentially important limit to the precision of 1 s in bottle lifetime measurements.

## THE APPARATUS

**FIG. 1.**
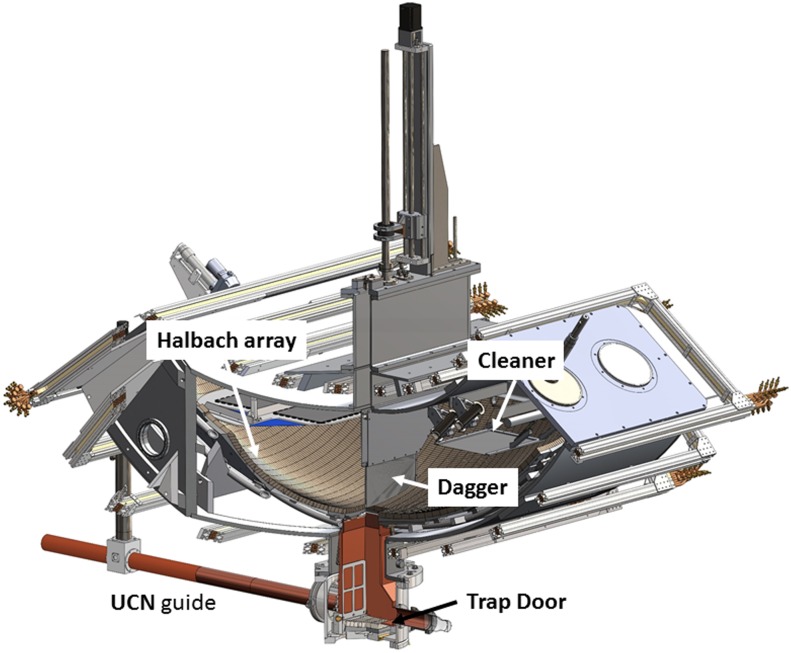
Cross sectional view showing the detector, the actuator, and the UCN trap. The locations of the dagger, cleaner, and trap door are shown. The dagger is in its lowest (counting) position and the trap door is in its loading (lowered) position.

**FIG. 2.**
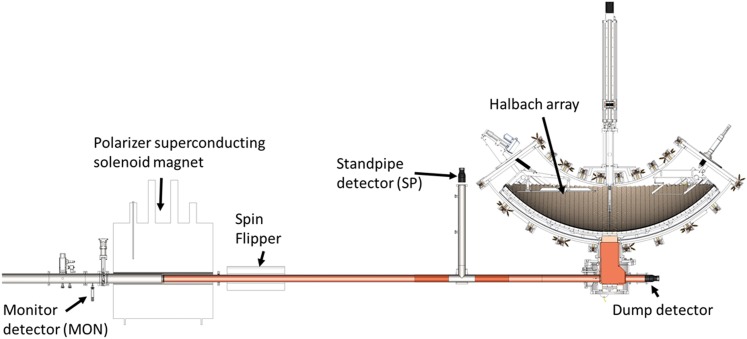
Schematic layout of the UCN beam line showing the monitor detector locations relative to the trap.

A cutaway view of the trap is shown in Figure 1 and a schematic layout of the beam line is shown in Figure 2. The detector discussed here is shown in its lowered position. A storage measurement cycle consists of loading UCN through a removable section at the bottom of the trap (trap door shown in its lowered position in Figure 1), cleaning neutrons with the cleaner lowered to a height of 40 cm above the bottom of the trap, closing the trap door to store neutrons, raising the cleaner and storing neutrons for a variable holding time, and finally lowering the detector (dagger) to count neutrons. The cleaner is a horizontal surface of a neutron absorbing material with a small negative potential. (In this case ^10^B on a ZnS substrate is the same material as the dagger.) Neutrons with enough energy to reach the cleaner are expected to eventually cross the cleaner surface and be absorbed. Monte Carlo calculations suggest that some nearly closed orbits can have long time constants. Later we describe how we measure the corrections due to quasi-bound UCN, which are not effectively cleaned.

UCN are provided by the Los Alamos UCN source^20^ at the Los Alamos Neutron Science Center (LANSCE). This is a spallation-driven solid-deuterium UCN source. The 800 MeV proton beam, which is used to produce neutrons, was on only for the loading period. This results in a low background environment for UCN counting.

A lifetime measurement consists of a sequence of measurements using a short holding time (e.g., 10 s) and a long holding time (e.g., 1410 s), from which the normalized number of UCN is obtained. Typically approximately 15 000 cleaned neutrons are detected in the trap at the short holding time. The statistical precision obtained in the lifetime from a single run pair (∼1 h) is about 12 s.

## THE DETECTOR

UCN were detected using commercial ZnS(Ag) screens,^21^ with 3.25 ± 0.25 mg/cm^2^ of phosphor, coated with 20 ± 5 nm of boron enriched to 95% ^10^B that was applied by vacuum evaporation. The thickness was monitored with an *in situ* quartz microbalance and a sapphire witness plate. The thickness was chosen as a tradeoff between UCN efficiency and light collection efficiency. The maximum energy of 38 neV for cleaned UCN stored in the trap was set by the vertical position of the cleaner. The thinner ^10^B coating provided high efficiency and several times more light than ∼120 nm coatings.

The UCN properties of the exposed materials of the detector are listed in Table I. The Fermi potential for neutrons is given by

$$V_{F}=\sum_{i}{\frac{2\pi \hbar }{m}}^{2}N_{i}a_{i},$$ (1)

where *m* is the neutron mass, *a*_*i*_ is the neutron coherent scattering length, and *N*_*i*_ is the material number density for the i-th constituent. The lifetime for neutron absorption in the material is

$$\tau_{A}=\frac{1}{\sum N_{i}\sigma_{Ai}\text{v}},$$ (2)

where *σ*_*Ai*_ are the neutron absorption cross sections and *v* is the UCN velocity. The cross sections are proportional to the inverse of the neutron velocity ($\sigma_{Ai}\propto 1/\text{v}$), and therefore the lifetime *τ*_*A*_ is independent of v.

**TABLE I.** UCN properties of the detector materials. V_Fermi_ is the surface potential and t is the adsorption time for UCN in the material.

Material	V_Fermi_ (neV)	Absorption time (ns)
^10^B	−3.7	8.4
Al	54.7	3.3 × 10^5^
ZnS	75.7	1.1 × 10^5^
Acrylic	27.6	2.4 × 10^5^
Polyimide	91.2	2.8 × 10^5^

As shown in Table I, all of the materials used in the detector assembly other than ^10^B and acrylic have positive *V*_*F*_ larger than the trap potential, so the UCN are expected to reflect from these materials with an absorption coefficient expected to be in the range of several times 10^−4^/reflection, typical of most materials. It should be noted that losses on reflection are likely to be an order of magnitude higher than this estimate because of the upscatter contribution to the cross section.^22^ However, this material is above the volume of the trap during storage of neutrons. The manufacture of the screen ensures that there is not much exposed acrylic. Scanning electron microscope images of the ZnS surface are shown in Ref. 23.

The reflection coefficient from the imaginary potential of the ^10^B can be significant^23^ requiring multiple bounces for detection and lengthening the collection time. The effect of the surface roughness of the screen, which may reduce the reflection, has not been quantified. Absorption on the other materials is negligible, even if several bounces are required for UCN to be absorbed. In its lowered position, the dagger subtends 40% of the area of the mid-plane. Typical orbit times in the trap are in the order of 1 s. The time constant of detection in a single bounce should be ∼2.5 s. The measured time constants of ∼6 s suggest that reflections lengthen the detection times.

Since UCN absorption on materials other than ^10^B is negligible, the spectral and time dependence of UCN detection can be accounted for by using the detection time to correct for systematic effects due to the coupling between phase space evolution and detection.

**FIG. 3.**
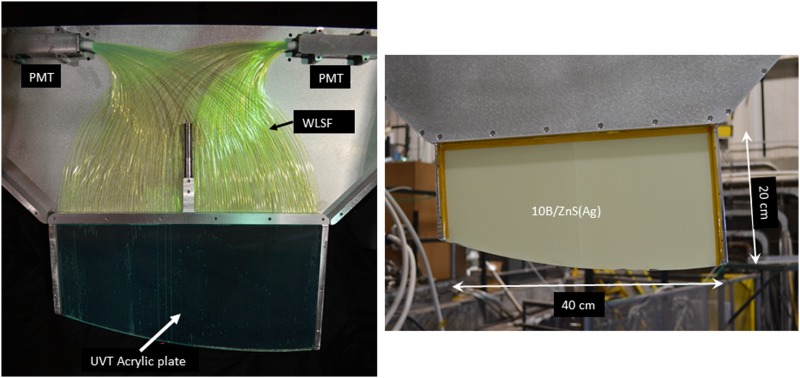
Photographs of the detector. (Left) During assembly, showing the phototube housings (PMT), the wave length shifting fiber light (WLSF) transport. (Right) Assembled detector.

Neutrons are captured by the ^10^B + n→α + ^7^Li(0 MeV),^7^Li(0.48 MeV) reaction with its large positive Q-value of 2.79 MeV and 2.31 MeV for the ground state and the first excited state of ^7^Li, respectively. The back-to-back correlation of the energetic charged particles ensures that at least one will stop in the ZnS(Ag) screen, producing scintillation light. This light is read out using an array of Kuraray Y-11(200) wave length shifting fibers, WLSFs, glued into an ultra-violet transmitting acrylic plate. The screen is fastened to the acrylic plate with optical epoxy. The fibers were glued into a set of 1 mm wide, 1 mm deep, 2 mm spaced grooves machined into a 3 mm thick plate that was backed by another 3 mm thick plate without grooves. Alternate fibers were directed into one of two photomultiplier tubes (PMTs). Photographs of the detector (dagger) are shown in Figure 3.

Some of the light produced in the ZnS(Ag) enters the WLSF, is shifted from blue to green, and is captured and transmitted to the phototubes by the fibers. By comparing the light output of the ZnS(Ag) measured with a phototube from a bare screen illuminated with a ^148^Gd(3.27 MeV) α-particle source with the light output measured for UCN absorption events in the dagger, we estimate the total photon detection efficiency to be 0.9% ± 0.3%.

The dagger was mounted on a linear vacuum feed through that allowed it to be raised and lowered within the UCN trap. In this way, UCN could be counted at various heights in the trap. The height resolution is limited by the contour on the bottom of the detector, which was designed to allow the detector to conform to the curved bottom of the trap.

As Leung *et al.* have discussed,^24^ moving a surface in a neutron trap can heat the UCN above the trap potential. To first order the fraction of neutrons that is heated is independent of storage time, so heating during counting has no effect on the lifetime measurement. Although quasi-trapped UCNs can be created by mechanical heating when the dagger is withdrawn from the trap, if it is used for cleaning, the corrections described below account for this.

**FIG. 4.**
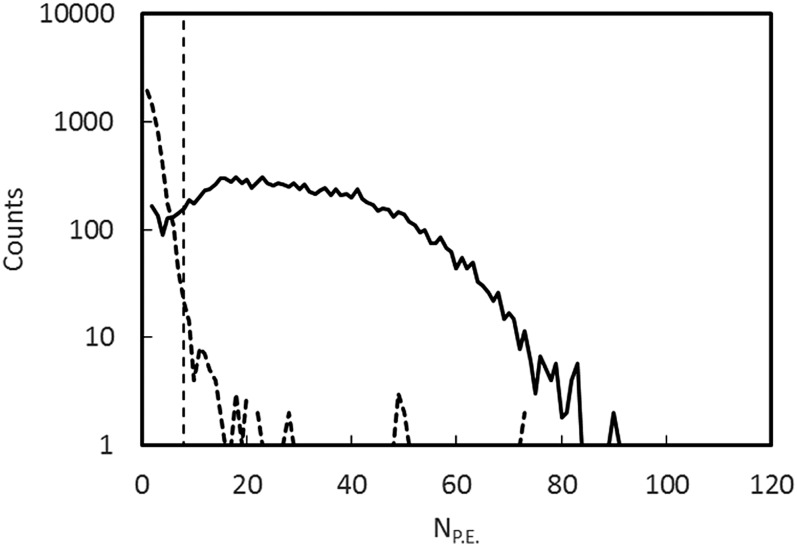
Spectra of the number of pulses (PE) detected for UCN + background events (solid line) and background events (dashed line). The vertical dashed line is PE = 8, the threshold chosen to define a UCN event. The normalizations are arbitrary.

Individual photo-electron signals from the photomultiplier tubes were amplified by a factor of ten in a fast amplifier and discriminated with a 0.5 photo-electron threshold. The resulting logic pulses with a width of 20 ns were digitized using a multi-channel scalar^25^ with a clock period of 0.8 ns. This allowed the summed number of photon pulses and coincidences between the two photomultipliers to be constructed in software. Because of the long mean decay time of the ZnS light emission, the summed number of resolved pulses provides an estimate of the energy deposited in the ZnS. A plot of the number of resolved pulses, labeled as photo-electrons (PEs), is shown in Figure 4, for both UCN and background events. For the purpose of forming this plot, events during the holding time were considered background and events during counting were considered UCN events. A coincidence within 100 ns was required between the two phototubes to define the start of an event. The number of photoelectrons in the event was obtained by counting pulses from both tubes until no new pulse arrived for a time greater than a looking time parameter, equal to 4 us for the analysis presented in this paper.

**FIG. 5.**
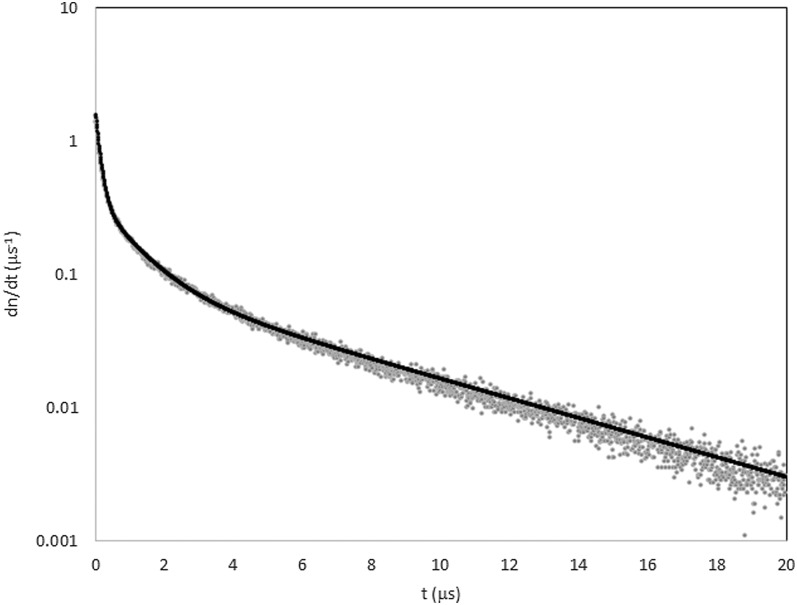
Detector pulse shape (grey points) and three-exponential fit (black curve).

The pulse time distribution of the light from events in the detector was measured by creating a histogram of the pulses from a set of events as a function of time after the first pulse detected for each UCN + background event. The normalized results and a fit using three exponentials are shown in Figure 5. The fraction of the light in each exponential was 0.18, 0.29, and 0.53, and the time constants were 0.134, 1.06, and 5.90 *μ*s, respectively. The amount of light in the short time constant component was sufficient to provide high efficiency coincidence counting of UCN.

**FIG. 6.**
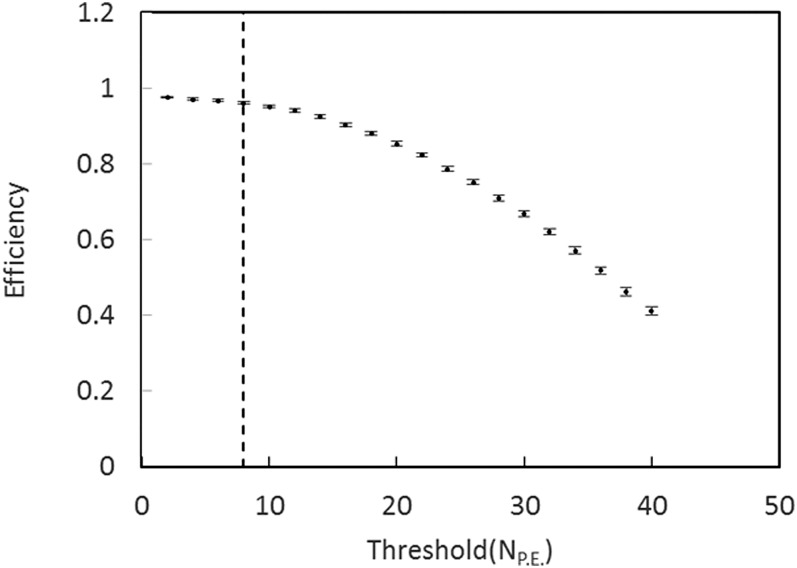
Efficiency as a function of the minimum number of PE required for an event. The dashed line is drawn at PE = 8. From Figure 4 it can be seen that the higher threshold results in a large decrease in the sensitivity to background with little loss in efficiency.

The efficiency of the dagger for light producing events was measured by mounting two 5-cm diameter phototubes above the trap with 7.5 cm diameter and 7.5 cm focal length Fresnel lenses to image light from one side of the dagger onto the photomultiplier tubes. A coincidence between these phototubes, DM, was used to tag UCN events from the adjacent side of the dagger. The dagger efficiency was calculated using UCN events when the dagger was lowered in to the trap, the unloading peak, as

$$eff=\frac{\text{Dagger}\cdot \text{DM}}{\text{DM}},$$ (3)

where the · designates a coincidence and Dagger are UCN events detected by the dagger. Equation (3) assumes that the Dagger and DM are independent. The inefficiency estimate does not include events that are absorbed but produce little or no light. The efficiency as a function of the threshold in terms of PE is shown in Figure 6. The efficiency for PE = 2, the minimum, is 0.976(2), and for PE = 8 the efficiency is 0.961(3).

The motion of the dagger with a velocity of ∼1 m/s is likely to upscatter some UCN that may escape the trap. The effects of dagger upscattering and inefficiencies remove the same fraction of UCN at long and short holding times so these effects do not affect the lifetime measurement. Higher order effects due to phase space evolution coupling to the inefficiencies and upscatter are small.

## CLEANING

During commissioning, we made the first lifetime measurement with the dagger described above. The sequence for these measurements was to fill the trap for 150 s and clean the trap for 200 s, with the cleaner down (38 cm above the bottom of the trap) for both operations. The area of the cleaner was 0.23 m^2^, 11% of the 2.04 m^2^ of the area of the spiric section through the trap at the cleaning height of 38 cm above the bottom.

At the end of the cleaning time, the cleaner was raised for the storage and counting parts of the run cycle. At the end of the storage time, the dagger was lowered to 1 cm from the bottom of the trap and the remaining UCN were counted for 100 s. At the end of the counting time, the trap door was opened and post-counting remaining neutrons were drained into an *ex situ* detector, the Dump detector shown in Figure 2. No evidence of post-counting remaining neutrons was observed in the Dump detector. Since the efficiency of the dump detector is estimated to be 25%, this measurement provides a highly sensitive test of the assumption that all of the neutrons are absorbed on the dagger. Surviving fractions as small as a few 10^−3^ should be easily observed.

The lifetime of neutrons in the trap was determined by counting the neutrons remaining after two different storage times. The initial number of neutrons loaded into the trap was determined by calculating yields normalized using two different monitor detectors (of five installed). The primary monitor, the standpipe detector (SP), was mounted at an elevation of 50 cm above the bottom of the trap on a tee in the UCN guide before the trap (see Figure 2), so it measured neutrons with energies above the maximum storable neutron energy of the trap. The second monitor detector, MON, was mounted near beam elevation and measured the incident UCN flux through an 8 mm diameter hole in the UCN guide near the biological shield wall. All of the monitors consisted of ^10^B-ZnS-PMT detectors described in Ref. 23.

The loading time constant was approximately 60 s and the trap was loaded for 150 s to reach approximate saturation. The normalization was obtained by convolving the SP rate with an exponential with a time constant of 60 s with respect to the time the trap door was closed. The SP detector was chosen as the primary monitor because of its higher counting rate and better statistics. The ratio of MON to SP decreased with time. Because the SP detector was elevated with respect to the MON detector this suggests that the fraction of faster UCN increased with beam exposure time. The ratio was increased by melting and refreezing the solid deuterium converter. This degradation was most likely caused by radiation damage to the solid deuterium crystal.

A linear correction which was a function of T=MON/SP was applied to correct for these spectral changes. The yields were calculated as:

$$Y_{S,L}=\frac{N_{S,L}}{\int_{-150}^{0}SP\left(t\right)e^{\frac{t}{60}}dt}\left(1+a\frac{T-\bar{T}}{\bar{T}}\right),$$ (4)

where N is the raw number of detected neutrons remaining in the trap at the end of the storage time (the subscript *S*, *L* denote short and long holding times, respectively), *a* is a constant that was fitted to minimize the sum of root mean square (RMS) of the long and short yields for each set of runs, t = 0 is the time relative to the time at which the trap door was closed and $\bar{T}$ is the average value of *T* for the data set consisting of multiple S,L pairs of runs. The spectral correction varied by ∼20% over a weekend of beam exposure. Alternating short and long holding time runs resulted in a high level of cancelation in this correction.

The lifetime of neutrons in the trap is then given by:

$$\tau =\frac{t_{l}-t_{S}}{\text{ln}\left(\frac{Y_{S}}{Y_{L}}\right)},$$ (5)

where *t*_*L*_, and *t*_*S*_ are the long and short holding times, respectively.

**FIG. 7.**
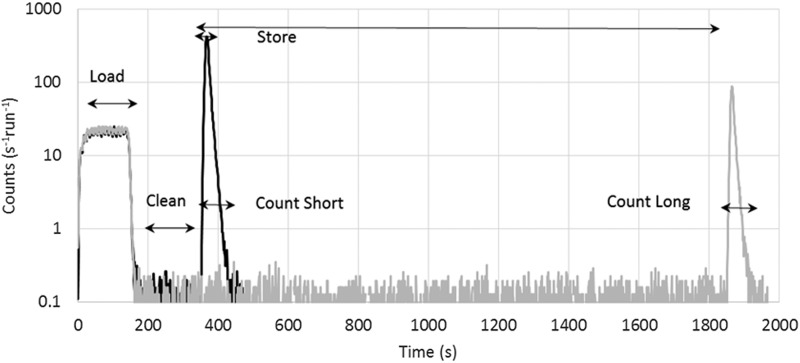
Summed short and long storage time distributions from the first τ_n_ data set.

The first data set consisted of 45 short (10 s) and 32 long (1510 s) long holding time runs. The summed time distributions of UCN + background events from this data set are shown in Figure 7. The lifetime from these data was found to be 858.4 ± 3.5 (stat.) s.

**FIG. 8.**
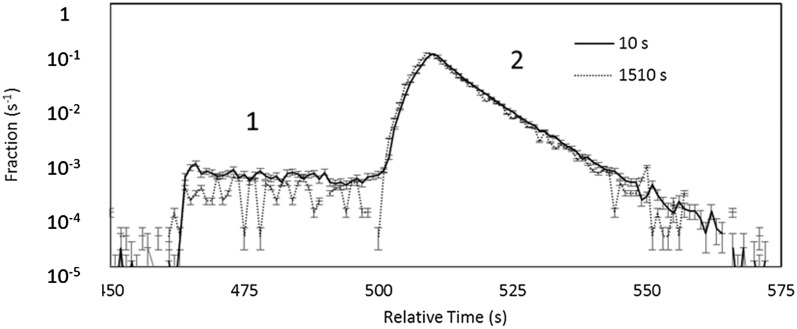
Overlay of the short and long, background subtracted, two step unloading time distributions for two step counting. The long holding time spectrum has been offset in time to line up with the short holding time spectrum, and both have been normalized by their integrals. Counting groups are labeled 1 and 2.

This resulted in the hypothesis that the UCN population in the trap was not sufficiently cleaned, and that quasi-bound neutrons were escaping during storage. In order to check this, the counting sequence was changed to lower the dagger in two steps, first to a position 37 cm from the bottom of the trap to count for 40 s (labeled 1 in Figure 8) and then to 1 cm from the bottom of the trap to count for 60 s (labeled 2 in Figure). The loading time and cleaning time for these data were 150 s and 300 s, respectively. The normalized time distributions, shown in Figure 8, show a shorter lifetime for the first counting group than for the second. The lifetime obtained by integrating the first 40 s of the counting time spectra from the first, higher energy, group was 614 ± 23 s. The lifetime for the second group was 880 ± 5 s after fitting and subtracting the contribution from the tail of first group. This contribution was calculated by fitting the first group data with an exponential and correcting its time constant for the neutron lifetime to extrapolate the contribution of these neutron to the second counting group, assuming that these neutrons were counted with a short time constant in the lower dagger position. A single exponential was used. There is no statistical evidence for the need for multiple exponentials. We have assumed the time constant for this correction is independent of holding time. This measurement both showed the cleaning to be insufficient and provides a correction method.

**FIG. 9.**
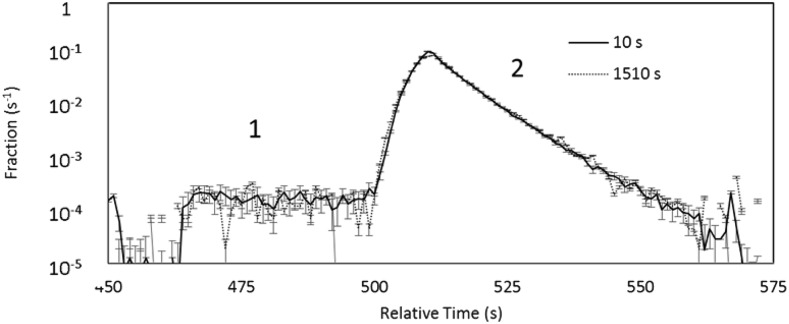
Same as Figure 8 but with dagger cleaning.

A third measurement was performed by lowering the dagger to the upper counting position during the loading and cleaning (dagger cleaning), raising it entirely out of the trap during the storage period, and then doing two step counting of the remaining neutrons. The time distributions of long and short storage runs (normalized by the integral counts), overlaid in Figure 9, show fewer counts in the first counting group (1) by a factor of 2.3, and the ratio of long to short rates of the two groups are much closer to being equal. The lifetime from the first group is 779 ± 49 s and the corrected lifetime from the second group is 878.4 ± 4.1 s, demonstrating more complete cleaning of quasi-bound neutrons. All of these results are summarized in Table II.

**TABLE II.** Preliminary measured and corrected values of the neutron lifetime. A. One step counting. B. Two step counting. C. Two step counting with dagger cleaning.

	Raw		Cleaning		Vacuum		Corrected	
Set	τ_measured_	Δτ_measured_	τ_correction_	Δτ_correction_	τ_correction_	Δτ_correction_	τ_n_	Δτ_n_
	s	s	s	s	s	s	s	s
A	858.4	3.6	15.4	3.1	0.4	0.1	874.2	4.7
B	862.8	5.7	17.4	1.5	1.6	0.5	881.8	6.0
C	876.5	4.0	1.9	0.9	0.9	0.3	879.3	4.1
						Average	878.1	2.8
						X^2^/dof	0.58	

Although dagger cleaning reduced the lifetime correction for quasi-trapped neutrons to 1.8 ± 0.8 s, this is still large compared to the goal of a 1 s counting statistics limited measurement. In order to further study energy distribution the trapped UCN a 4-step counting sequence was used, with dagger positions of 37, 25, 13, 1 cm from the bottom of the trap.

**FIG. 10.**
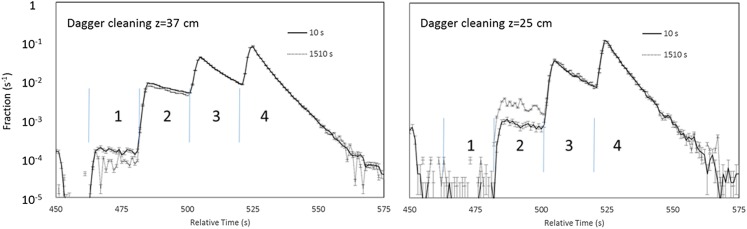
Overlay of the short (solid line) and long (dashed line), background subtracted four step unloading time distributions for 300 s cleaning using both the cleaner and the dagger, with the dagger lowered to 37 cm (left) and 25 cm (right). The numbers, 1-4, label the counting positions.

We have used four-step counting to study other cleaning conditions. Since these data were part of production data taking, they were blinded, so lifetime results are not presented here. Some results are shown in Figure 10 to illustrate features of cleaning. The spectrum on the left shows a significant population of UCN in the counting group 1. The data on the right were cleaned using the same cleaner cycle as those on the left, but with the dagger lowered to 25 cm (well below the height of the lowered cleaner of 38 cm) for the loading and cleaning times. In this mode there were negligible counts measured in the counting group 1, at a height of 37 cm. The relative number of counts in the counting group 2 is observed to increase with holding time. This is because the cleaning apparently reduced the population of neutrons in the region of phase space of orbits that are counted in the second dagger position. This creates a hole in the phase space which heals as neutrons redistribute in phase space at longer holding times. Because of the short counting times, this comparison demonstrates that the active dagger detector allows more effective probing of the dynamics of the trapped UCN than can be obtained in storage experiments with traditional *ex situ* detectors.

We found debris from copper tape in the bottom of the trap that was pulled into the trap by the trap door. The debris appeared between the data shown at the left in Figures 10 and 9. The debris reduced the effective depth of the trap and explains the difference in cleaning efficiency in the two data sets.

## PRELIMINARY LIFETIME RESULTS

**FIG. 11.**
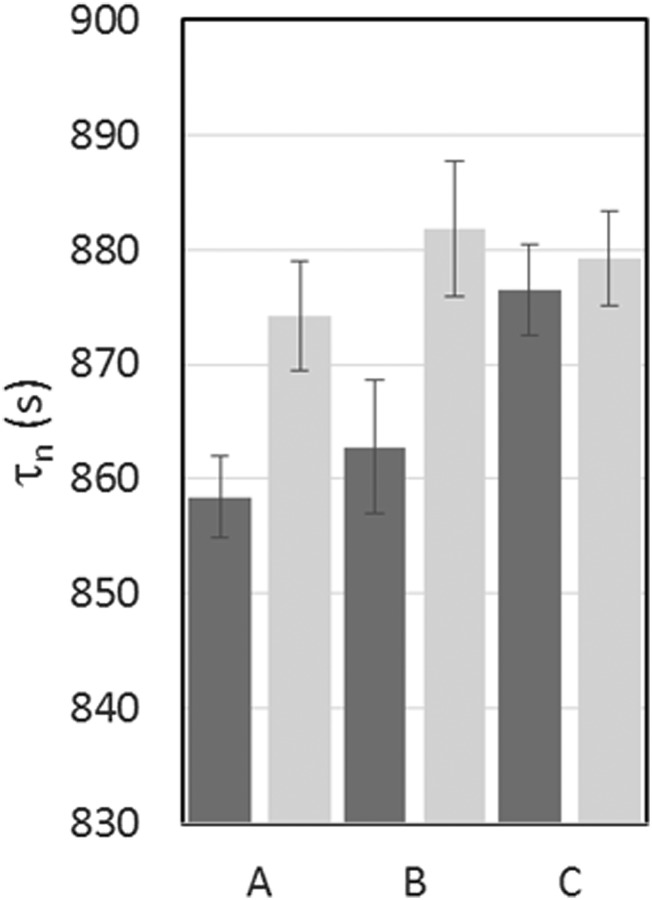
A plot showing the measured lifetimes (dark grey) and corrected for marginally trapped UCN (light grey) for three different sets of runs: (A) one step counting, (B) two step counting, (C) two step counting with dagger cleaning.

The neutron lifetimes obtained from the three counting conditions described in the previous section are summarized in Table II and plotted in Figure 11. The live time corrected yields were calculated using Equation (4). The data were analyzed in time adjacent pairs, and the lifetimes were calculated according to Equation (5). Three corrections are applied to these lifetimes: first for the measured effect of uncleaned neutrons, second for the residual pressure in the trap, and third we used the centroids measured time spectra to determine the holding time. The cleaning correction for this data set was obtained from several doublets of runs with 4 step dagger counting and 200 s cleaning. The pressure correction was made using a calibrated cold cathode gauge to measure the pressure, an RGA to measure the mass spectrum of the gas and measured cross sections^26^ to calculate the velocity independent UCN lifetime due to losses on the residual gas in the trap. Finally, the effects of control timing errors and phase space evolution were accounted for by using the centroid of the long and short counting times to determine *t*_*l*_ − *t*_*s*_. The corrections to the holding time were 2.5 ± 0.5 s for data set A and B, dominated by control error, and 0.8 ± 0.2 s for data set C dominated by phase space evolution. Here the uncertainties are statistical. Because of the short counting time, the phase space evolution correction and its uncertainty are small. Remaining systematic uncertainties are listed in Table III.

**TABLE III.** Estimated systematic uncertainties not included in Table II.

Effect	Upper bound (s)	Direction	Current eval.	Method of characterization
Depolarization	0.01	+	Calculated	Theory
Microphonic heating	0.1	+	Simulated	Accelerometer studies
Dead time/pileup	0.5	±	Simulated	Coincindence studies
Time dependent background	0.1	±	Measured	Measurements
Gain drifts	0.2	±	Measured	Measurements
Phase space evolution	0.2	±	Measured	Measurements
Total	0.6		(Uncorrelated sum)	

One significant systematic uncertainty is due to dead-time/pile-up. The dead time correction to the short holding time runs is larger than for the long holding time runs. The dead time is calculated as the width the photon counting time is opened for each event. Monte Carlo simulations show that this slightly overestimates the dead time because an event within this gate can occasionally generate a coincidence after the end of the gate and be counted. We have estimated the size of the effect to be as large as 0.5 s. The dead time algorithm will be improved in the future.

The cleaning correction for the set of data taken using the dagger to augment the cleaner is relatively small (1.9 s) when compared to the sets where only the cleaner was used for cleaning. Nevertheless, the correction is observed to bring all three sets into statistical agreement, see Figure 11, supporting the conclusion that the dagger effectively measures the correction. The agreement between the three data sets argues against accidental cancellation between phase space evolution and marginally trapped neutrons escaping the trap. Subsequent to these measurements a larger area cleaner was installed to improve the cleaning. In Table III we present estimates of the remaining systematic uncertainties.

## CONCLUSION

We have described a new method for *in situ* counting of neutrons in a magneto-gravitational trap. The dagger detector allows the systematic correction for insufficient cleaning to be measured and the lifetime data to be corrected. The counting time using this detector is comparable to the uncertainty in the lifetime, ensuring that corrections due to phase space evolution on the neutron holding time can be measured to relatively high precision. Further, these measurements have led to the implementation of a more effective cleaner with a much larger surface area. This cleaner will be used in subsequent experimental campaigns.

Neutron lifetimes were extracted from three data sets that were taken using different cleaning conditions. These data sets resulted from our commissioning runs and were never blinded. The lifetimes extracted from the three sets of data are in agreement, and they give an average neutron lifetime τ_n_ = 878.1 ± 2.8 ± 0.6 s, in good agreement with previous bottle lifetime measurements but in disagreement with the beam measurements. The method described here will be applied to a blinded dataset with higher statistical sensitivity, and any remaining potential sources of systematic uncertainty will be quantified. Our definitive lifetime value will come from a future blinded analysis of later data sets.
